# Spontaneous ureteric rupture, a reality or a faux pas?

**DOI:** 10.1186/s12894-016-0158-2

**Published:** 2016-07-07

**Authors:** Gaurav Aggarwal, Samiran Das Adhikary

**Affiliations:** Department of Urology, Apollo Hospital, Bhubaneshwar, 751005 Odisha India

**Keywords:** Ureteric rupture, Spontaneous, DJ stenting, Case report

## Abstract

**Background:**

Rupture of the urinary collecting system with or without any perinephric extravasation is an extremely rare occurrence and usually known to occur following an obstructive pathology.

Spontaneous or non-traumatic rupture, in the absence of any distal obstruction, though reported in literature, is not yet a proven entity and needs to be distinguished from physiological forniceal rupture, to validate its occurrence. Our case illustrates that spontaneous ureteric rupture does exist and requires a high level of vigil for prompt diagnosis and early simple management.

**Case presentation:**

A 65 year old non diabetic gentleman presented with a 2 day history of right sided severe abdominal pain with no history of any prior trauma, surgery, urinary retention or calculus disease. His ultrasound whole-abdomen was suggestive of increased liver echogenicity, but his contrast enhanced CT scan (CECT) documented a ureteric rupture, with leakage of contrast from the upper ureters, well away from the renal pelvis

He was promptly managed with cysto-ureteroscopy, retrograde pyelography (RGP) and double-J (DJ) stenting. His post operative course was uneventful and he was discharged on the second post operative day, without event.

An RGP at 6 weeks of follow up showed no contrast extravasation from the ureter and his DJ stent was removed without event.

**Conclusion:**

Spontaneous ureteric rupture, in the absence of any inciting cause, is an entity which exists and is easily manageable, once diagnosed timely. Thus, the need to maintain a high index of vigil, in order to identify this clinically entity at the earnest, institute prompt treatment and hence ensure that a “spontaneous” rupture, doesn’t become a “faux pas” in the true sense of the word.

## Background

Urinary collecting system rupture with or without perinephric extravasation is an extremely rare occurrence and usually thought to occur following an obstructive pathology.

Spontaneous or non-traumatic rupture [1, 2], though reported in literature, is not yet a proven entity and needs to be distinguished from physiological forniceal rupture, to validate its occurrence.

Our case illustrates that spontaneous ureteric rupture exists and requires a high level of vigil for prompt diagnosis and simple management.

## Case Presentation

A 65 year old non diabetic, non-alcoholic gentleman presented with a 2 day history of right sided severe abdominal pain with no prior trauma, surgery, urinary retention or calculus disease.

Clinically, he was vitally stable, apart from mild tachycardia (pulse rate 92 beats/min). On local examination, there was no abdominal or costo-vertebral tenderness and rest of his abdomen was unremarkable, however his pain was seemingly out of proportion to these examination findings. His serum urea/creatinine, liver function tests, serum amylase/lipase as well as all other biochemical tests were within normal limits.

His ultrasound whole-abdomen was suggestive of increased liver echogenicity, with no evidence of any abnormality to the kidneys, ureters, bladder as well as no intra or retroperitoneal fluid collection, and he was being evaluated along the lines of a liver abscess.

**Fig. 1:**
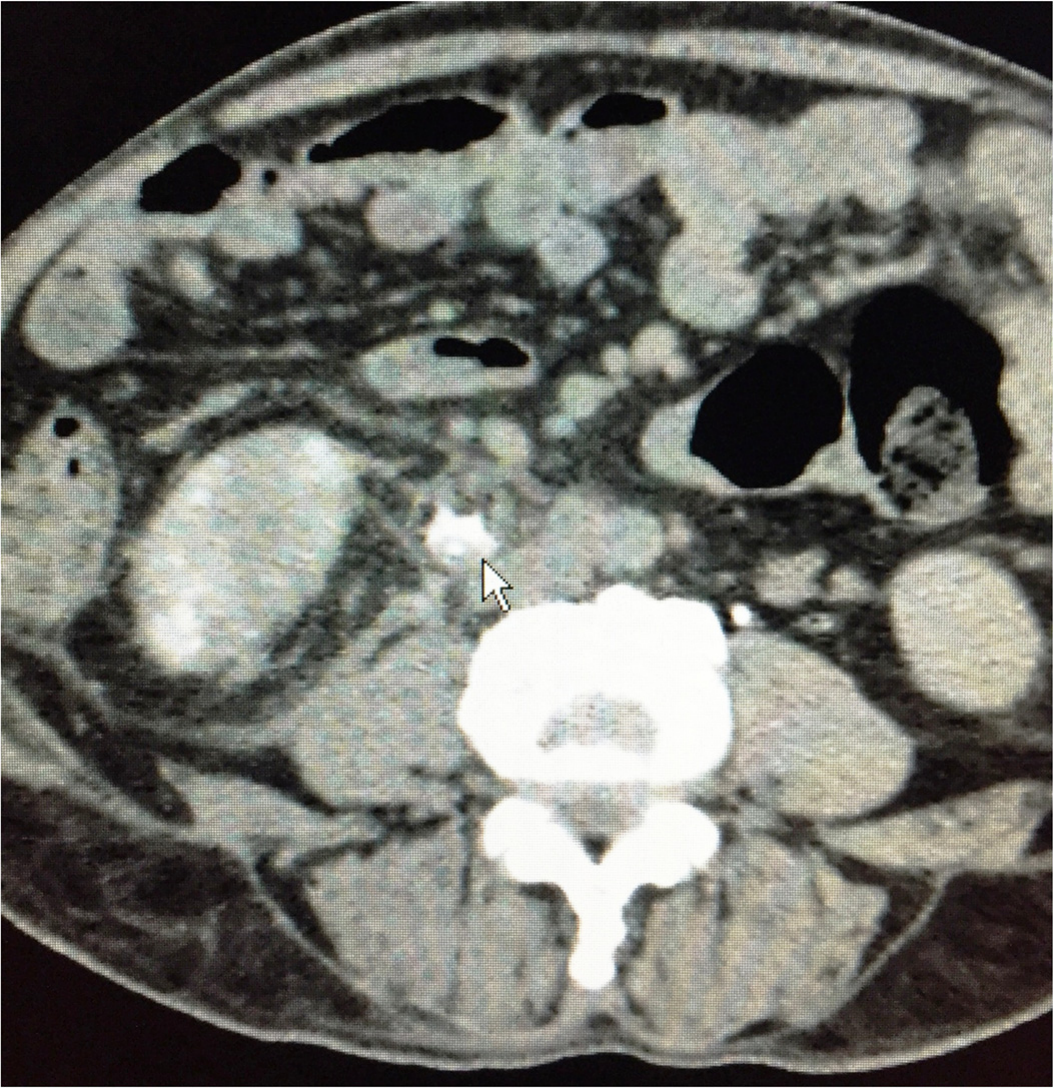
CECT with arrow demonstrating contrast leak from the upper ureter, well away from the fornix

However, a CECT scan when done, reported a ureteric rupture with leakage of contrast from the upper ureter, well away from the renal pelvis, at the level of the 2^nd^ lumbar vertebra (Fig. 1).

**Fig. 2:**
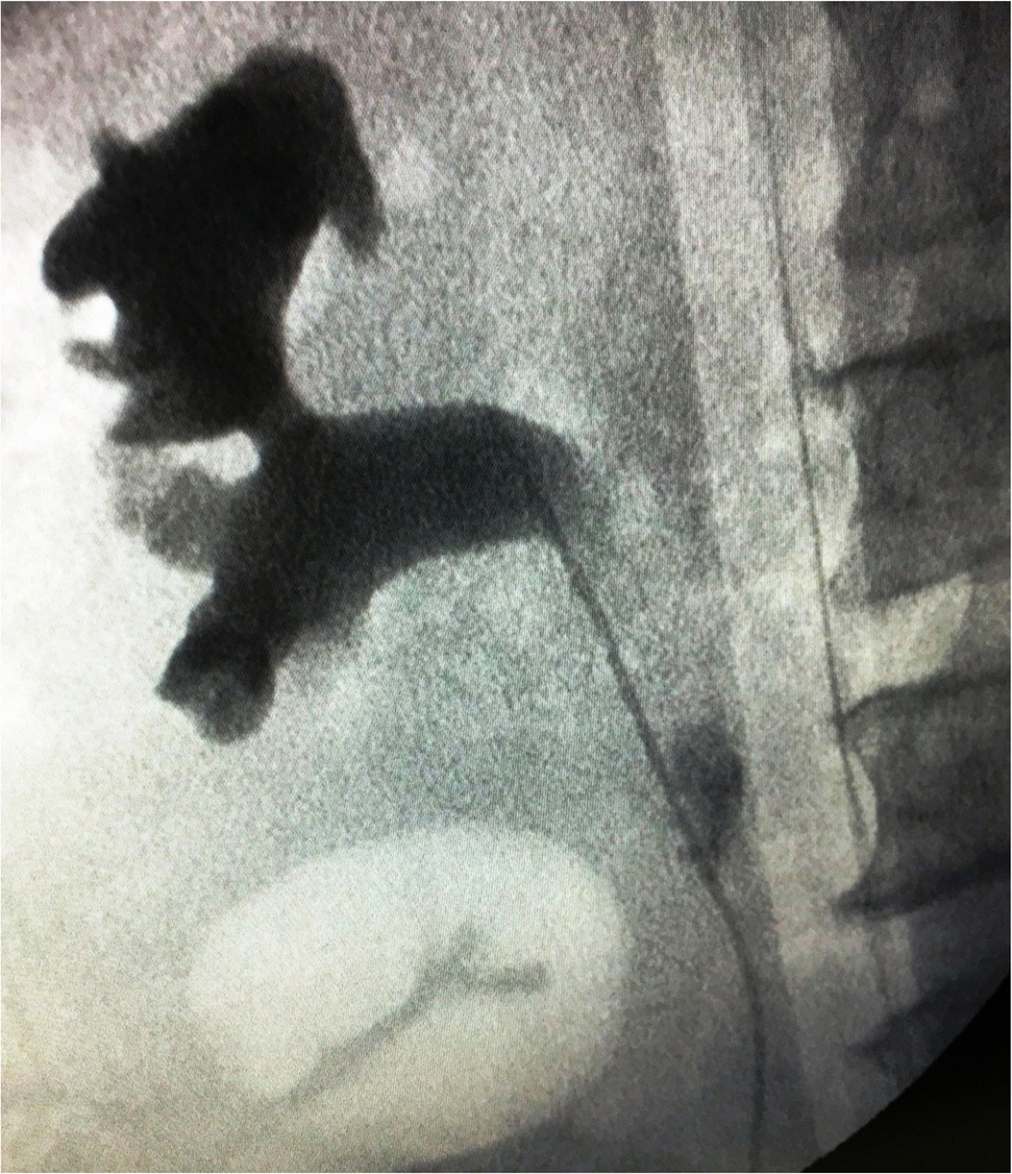
Intra-operative RGP, demonstrating contrast extravasation from upper ureter

He was promptly taken up for cysto-ureteroscopy, retrograde pyelography (RGP) and DJ stenting. Intraoperative RGP demonstrated the upper ureteric rupture at a fair distance from the pelvis without cystoscopic evidence of any calculus or obstructive uropathy (Fig. 2).

He was managed via cystoscopy and placement of a DJ Stent (5Fr, No 26). His post operative course was uneventful and he was discharged on the second post operative day, without event.

**Fig. 3:**
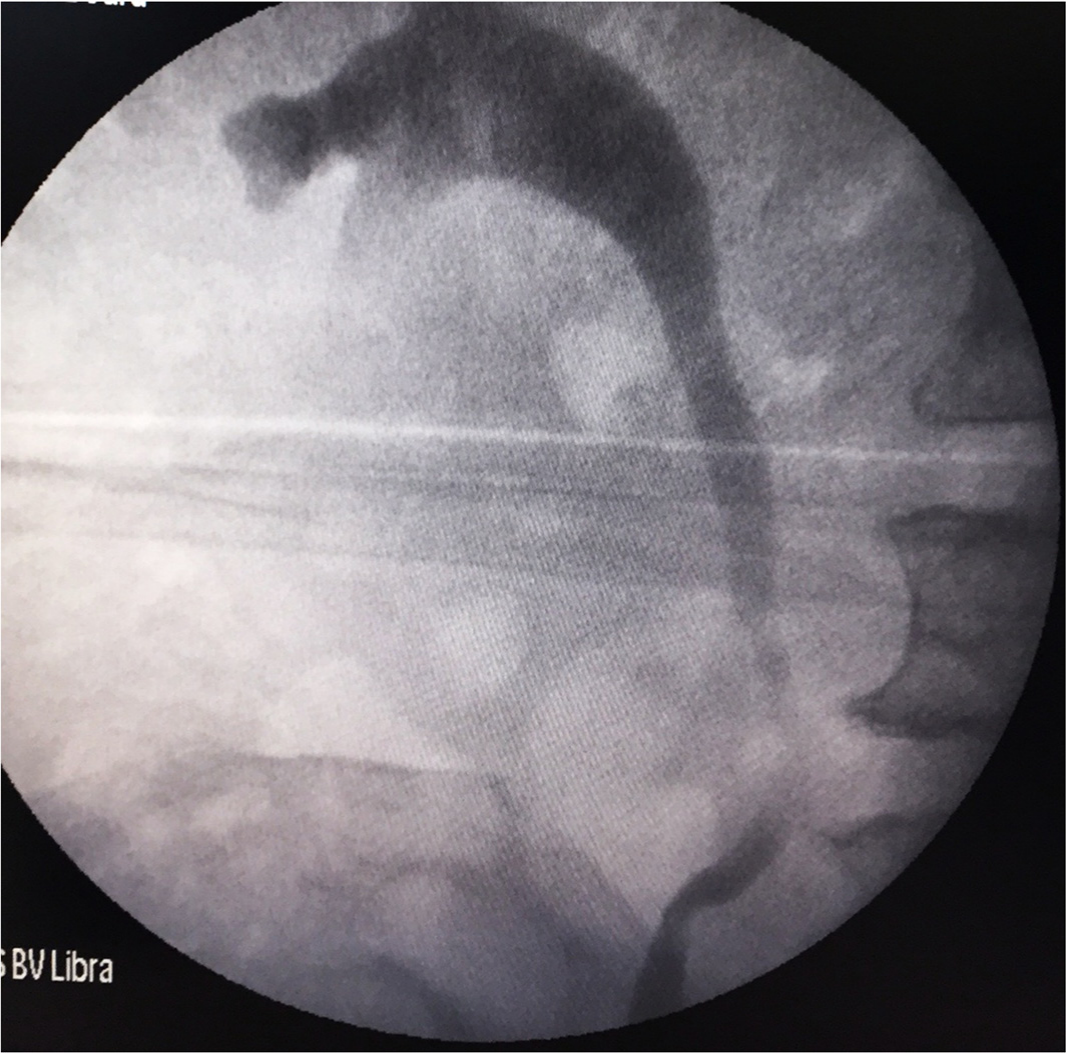
A follow up RGP at the time of stent removal, demonstrating the completely healed ureter (no contrast extravasation)

At 6 weeks of follow up, there was no contrast extravasation as confirmed via an RGP done during stent removal (Fig. 3).

The patient was discharged without event and advised to remain on 6 monthly follow ups, with imaging to be done as per his subsequent symptoms.

## Discussion

Ureteric rupture is in itself an infrequently encountered entity and a spontaneous rupture is even rarer [1].

Rupture of the ureter is usually expected to occur following trauma, which may be blunt, penetrating or even iatrogenic [1, 2]. Spontaneous or non-traumatic rupture, if at all, is considered to be secondary to some downstream obstruction by a calculus, papilla, stricture or via extrinsic compression [1, 2].

The symptom at presentation, mainly sudden, severe lower abdominal pain is usually not in sync with the clinical signs and may mimic an episode of acute appendicitis or diverticulitis [2].

Thus, the diagnosis frequently gets delayed leading to long term patient morbidity [2]. Initially, an excretory urography was considered as the imaging modality of choice, however, in the current era, a contrast enhanced CT scan (CECT) forms the mainstay for imaging with intraoperative retrograde urography (RGU) being the gold standard [1–3].

Koga et al. (11 cases) [4] and Stravodimos et al. (5 cases) [5] remain the largest series till date, having reported spontaneous peripelvic collections, of which only 4 of the cases in the work by Stravodimos et al. were not found to have any cause, or could be said as “spontaneous” ruptures.

On account of these few and interspersed reports and lack of specific guidelines, management strategies vary among surgeons, from endoscopic stenting to even open surgical repair [3–5]. There have been reports in literature documenting even percutaneous drainage, as well as conservative management with just antibiotics till clinical deterioration, but all of them suggest that a prompt intervention definitely reduces long term morbidity [6].

Prognosis remains excellent, once diagnosed early, and managed promptly, with immediate relief. On the contrary, a delay in instituting treatment may lead to serious consequences such as a perinephric or retroperitoneal collection, abscess formation and subsequently urosepsis [6].

Thus, our case illustrates that timely diagnosis and prompt management in the acute setting via simple DJ stenting is sufficient to allow complete healing and prevent long term complications.

## Conclusion

Spontaneous ureteric rupture, in the absence of any inciting cause, is an existing entity, easily manageable, once diagnosed timely.

As is said, *“what the mind knows is what the eyes see”.* This statement typically exemplifies the need to keep this clinical entity in mind, so as to prevent a “*spontaneous*” rupture from becoming a “*faux pas”* in the true sense of the word.

## Abbreviations

CECT, Contrast Enhanced CT Scan; RGP, Retrograde Pyelography; DJ Stenting, Double J stenting.
